# Outcomes of endoscopic retrograde cholangiopancreatography performed in the AM versus PM: does procedural timing matter?

**DOI:** 10.1093/jcag/gwae028

**Published:** 2024-08-26

**Authors:** Nasruddin Sabrie, Nikko Gimpaya, Kareem Khalaf, Maya Deeb, Wedad Mhalawi, Michael Meleka, Daniel C Tham, Ahmed H Mokhtar, Caleb Na, Sophia P Abal, Sharan B Malipatil, Sarang Gupta, Sechiv Jugnundan, Deiya Chopra, Rishad Khan, Natalia C Calo, Christopher W Teshima, Gary R May, Jeffrey D Mosko, Samir C Grover

**Affiliations:** Division of Gastroenterology, St. Michael’s Hospital, University of Toronto, Toronto, Ontario, Canada; Division of Gastroenterology, St. Michael’s Hospital, University of Toronto, Toronto, Ontario, Canada; Division of Gastroenterology, Department of Medicine, Scarborough Health Network, University of Toronto, Toronto, Ontario, Canada; Scarborough Health Network Research Institute, Toronto, Ontario, Canada; Division of Gastroenterology, St. Michael’s Hospital, University of Toronto, Toronto, Ontario, Canada; Division of Gastroenterology, St. Michael’s Hospital, University of Toronto, Toronto, Ontario, Canada; Division of Gastroenterology, St. Michael’s Hospital, University of Toronto, Toronto, Ontario, Canada; Division of Gastroenterology, St. Michael’s Hospital, University of Toronto, Toronto, Ontario, Canada; Division of Gastroenterology, St. Michael’s Hospital, University of Toronto, Toronto, Ontario, Canada; Division of Gastroenterology, St. Michael’s Hospital, University of Toronto, Toronto, Ontario, Canada; Division of Gastroenterology, St. Michael’s Hospital, University of Toronto, Toronto, Ontario, Canada; Division of Gastroenterology, St. Michael’s Hospital, University of Toronto, Toronto, Ontario, Canada; Division of Gastroenterology, St. Michael’s Hospital, University of Toronto, Toronto, Ontario, Canada; Division of Gastroenterology, St. Michael’s Hospital, University of Toronto, Toronto, Ontario, Canada; Division of Gastroenterology, St. Michael’s Hospital, University of Toronto, Toronto, Ontario, Canada; Division of Gastroenterology, St. Michael’s Hospital, University of Toronto, Toronto, Ontario, Canada; Division of Gastroenterology, St. Michael’s Hospital, University of Toronto, Toronto, Ontario, Canada; Division of Gastroenterology, St. Michael’s Hospital, University of Toronto, Toronto, Ontario, Canada; Division of Gastroenterology, St. Michael’s Hospital, University of Toronto, Toronto, Ontario, Canada; Division of Gastroenterology, St. Michael’s Hospital, University of Toronto, Toronto, Ontario, Canada; Division of Gastroenterology, St. Michael’s Hospital, University of Toronto, Toronto, Ontario, Canada; Li Ka Shing Knowledge Institute, University of Toronto, Toronto, Ontario, Canada; Division of Gastroenterology, St. Michael’s Hospital, University of Toronto, Toronto, Ontario, Canada; Division of Gastroenterology, Department of Medicine, Scarborough Health Network, University of Toronto, Toronto, Ontario, Canada; Scarborough Health Network Research Institute, Toronto, Ontario, Canada; Li Ka Shing Knowledge Institute, University of Toronto, Toronto, Ontario, Canada

**Keywords:** ERCP, adverse events, quality improvement

## Abstract

**Background:**

ERCP is a technically demanding procedure that carries a high cumulative adverse event (AE) rate of >10%. Identifying risk factors for adverse events is paramount. Procedure timing, as a surrogate for endoscopist fatigue, has been shown to influence key quality metrics in colonoscopy, but data on this relationship in ERCP is sparse.

**Methods:**

We conducted a retrospective cohort study of ERCP procedures performed by 5 experienced staff endoscopists, with or without advanced endoscopy fellow (AEF) involvement, from January 1, 2010 to December 1, 2020 at St Michael’s Hospital, Toronto, Ontario, a regional referral center for therapeutic endoscopy. The primary outcome was the difference in rate of selective deep, duct canulation between AM and PM procedures.

**Results:**

A total of 5672 ERCP procedures were eligible for inclusion. 2793 (49.2%) procedures were performed in the AM and 2879 procedures (50.8%) were performed in the PM. We found no significant difference in the rate of selective ductal cannulation between AM and PM procedures in the unadjusted (82.8% AM vs. 83.1% *P*-value = .79) or adjusted (OR = 0.98, 95% CI, 0.85-1.12, *P*-value = .72) analyses. We found no significant difference in the mean procedural duration or rate of perforation between AM and PM procedures. The rate of immediate bleeding was slightly higher in the AM cohort.

**Conclusion:**

In our single-center retrospective study, ERCP quality, including selective cannulation rates and immediate adverse events were not significantly different in procedures performed in the morning compared to those performed in the afternoon.

## Introduction

Endoscopic retrograde cholangiopancreatography (ERCP) is a technically demanding procedure that carries a high cumulative adverse event (AE) rate of >10%.^1^ These complications include post-ERCP pancreatitis (PEP), bleeding, perforation, and cardiopulmonary events.^1^

Key quality indicators include rates of such AEs as well as intraprocedural endpoints such as selective cannulation of ducts of interest.^2^

Identifying both, modifiable barriers to achieving these key quality indicators and risk factors for adverse events, is paramount. Procedure timing, as a surrogate for endoscopist fatigue, has been shown to influence key quality metrics in colonoscopy.^3–6^ For example, polyp and adenoma detection rates (ADR) have been found to be reduced in afternoon colonoscopies compared to morning colonoscopies.^5,6^ Similarly, completion rates and cecal intubation rates have been found to be lower in afternoon colonoscopies.^3,4^

There are sparse data on the relationship between procedural timing and outcomes in ERCP. Two prior studies on this topic, by Mehta et al. and Nelsen et al., did not identify any relationship between ERCP timing and cannulation success or adverse events.^7,8^ These studies were limited however, by a small sample size^7^ and lack of generalizability due to procedures being conducted with support from an anesthesia professional,^8^ respectively. Thus, to address these gaps, we performed a retrospective cohort study at a large ERCP referral center. St Michael’s hospital is a tertiary, academic hospital with an emphasis on advanced endoscopy training which will serve to generalize our findings to other teaching institutions.

## Methods

### Study design

We conducted a retrospective cohort study of ERCP procedures performed by 5 experienced staff endoscopists, with or without advanced endoscopy fellow (AEF) involvement, from January 1, 2010 to December 1^st^, 2020 at St Michael’s Hospital, Toronto, Ontario, a regional referral center for therapeutic endoscopy. We utilized a prospectively maintained institutional database containing clinical and procedural data of all ERCP’s performed during the aforementioned time frame [REB #23-110].

### Inclusion criteria

We included all diagnostic and therapeutic ERCPs performed in adult patients over the age of 18 during predefined daytime working hours of 8 AM to 6 PM. Procedures performed before 8 AM and after 6 PM were excluded from our study. We defined AM procedures as those that began between 8 AM and 12 PM, and PM procedures as those that began between 12 PM and 6 PM.

### Data collection

We collected baseline characteristics of patients undergoing ERCP including their age, gender, comorbidities, inpatient-status, and baseline medications. Procedural details were also captured including the method of sedation (conscious sedation or general anesthesia), the indication for the procedure, the involvement of trainees, selective cannulation rates, the performance of sphincterotomy, duration of the procedure, and adverse events.

### Outcomes

The primary outcome was the difference in rate of selective deep, duct canulation between AM and PM procedures. We chose selective duct cannulation as it has been highlighted as a key quality indicator for ERCP by the American Society for Gastrointestinal Endoscopy (ASGE) and American College of Gastroenterology (ACG).^9^ Secondary outcomes included procedural duration and the adverse events of intraprocedural bleeding and perforation. Intraprocedural bleeding was defined as any visible oozing or spurting of blood that lasted for longer than 30 s or required intervention. Perforation was defined as a visible full-thickness luminal defect visualized during endoscopy or radiographic evidence of a luminal defect and/or free intra-abdominal air immediately after the procedure. We did not include delayed bleeding, delayed perforation, or post-ERCP pancreatitis as outcomes as our database did not capture any health encounters outside of St Michael’s Hospital, and would thus have missed cases where patients presented to other hospitals.

### Statistical analysis

Statistical analysis was conducted using R (R Core Team 2022). Categorical variables were compared using the chi-square test of independence or Fisher’s exact test.^10^ Continuous variables were compared using *t*-tests or the Mann–Whitney-*U* test.^10^ The normality of a given variable was assessed using the Shapiro–Wilk’s test.^11^ All statistical tests were evaluated at a *P*-value of < .05. Multivariable logistic regression models were used to assess the impact of ERCP timing (AM or PM) on primary and secondary outcomes with adjustment for important clinical variables reported to be significant in prior literature^1,12^ and variables with significant differences between the 2 cohorts on univariate analysis. Subgroup analyses were conducted in procedures without AEF involvement and procedures in patients with altered anatomy.

## Results

### Baseline characteristics

A total of 5672 ERCP procedures were eligible for inclusion. Two thousand seven hundred and ninety-three (49.2%) procedures were performed in the AM and 2879 procedures (50.8%) were performed in the PM (Table 1). The mean age was 63.9 years (SD 17.7 years), and 2759 (48.6%) patients were males. The majority (93.3%) of ERCPs was performed on outpatients or inpatients referred-in from outside hospitals. Hypertension and diabetes mellitus were the most frequently reported comorbidities (32.0% and 20.2%). At baseline, 10.5% of patients were on antiplatelets, and 19.3% of patients were on anticoagulants. The most common procedural indication was for choledocholithiasis (44.6%).

**Table 1.** Baseline characteristics and procedure indications.

	Total (*n* = 5672)	AM (*n* = 2793)	PM (*n* = 2879)
Age (mean, SD)	63.9 (17.7)	63.7 (17.7)	64.0 (17.6)
Male	2759 (48.6%)	1345 (48.2%)	1414 (49.1%)
Inpatients	379 (6.7%)	134 (4.8%)	245 (8.5%)
**Comorbidities**			
Renal disease	254 (4.5%)	131 (4.7%)	123 (4.3%)
Liver disease	284 (5.0%)	146 (5.2%)	138 (4.8%)
Hypertension	1813 (32.0%)	944 (33.8%)	869 (30.2%)
Diabetes	1145 (20.2%)	592 (21.2%)	552 (19.2%)
**Baseline antithrombotic**			
Antiplatelets	595 (10.5%)	298 (10.7%)	297 (10.3%)
Anticoagulants	1093 (19.3%)	566 (20.2%)	527 (18.3%)
**Procedure indication**			
Bile duct stone	2531 (44.6%)	1253 (44.9%)	1278 (44.4%)
Pancreatic disease	465 (8.2%)	224 (8.0%)	241 (8.4%)
Stricture	382 (6.7%)	198 (7.1%)	184 (6.4%)
Cholangitis	84 (1.5%)	44 (1.6%)	40 (1.4%)
Other^a^	2210 (39.0%)	1074 (38.5%)	1136 (39.5%)

^a^Includes procedures performed for abnormal imaging, elevated liver enzymes, ampullar lesions, and biliary stent-related issues.

### Procedural details

The majority of the procedures, 94.7% in the AM group and 94.5% in the PM group had trainee involvement (Table 2). Less than 5% of patients in both groups had altered surgical anatomy (96.6% AM vs. 96.3% PM). Conscious sedation was used for 98.7% of patients in the AM vs. 98.6% in the PM group. A sphincterotomy was performed for 63.4% of patients in the AM group and 62.6% in the PM group.

**Table 2.** Procedural details, endpoints, and adverse event rates.

	AM (*n* = 2793)	PM (*n* = 2879)	*P* *-value*
**Procedural details**			
Clinical fellow as primary operator	2646 (94.7%)	2722 (94.5%)	*.75*
Normal anatomy	2698 (96.6%)	2773 (96.3%)	*.57*
Conscious sedation	2758 (98.7%)	2838 (98.6%)	*.58*
Sphincterotomy performed	1770 (63.4%)	1802 (62.6%)	*.54*
**Procedural endpoints** ^a^			
Selective ductal cannulation	2312 (82.8%)	2391 (83.1%)	*.79*
Duration (min)	33.7	33.0	*.29*
**Adverse outcomes** ^a^			
Immediate bleeding	141 (5.0%)	110 (3.8%)	***.03***
Perforation	7 (0.3%)	5 (0.2%)	*.53*

^a^Unadjusted outcomes.

### Primary outcome

**Figure 1.**
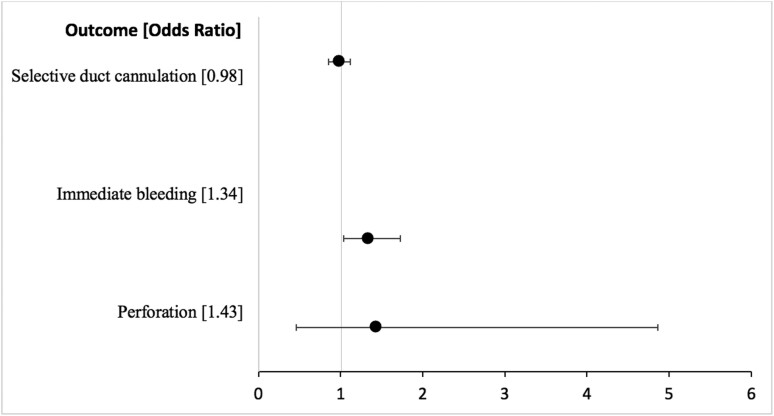
Adjusted odds ratio for deep cannulation and adverse outcomes: AM vs. PM.

We found no significant difference in the rate of selective ductal cannulation between AM and PM procedures in the unadjusted (82.8% AM vs. 83.1% *P*-value = .79) (Table 2) or adjusted (OR = 0.98, 95% CI, 0.85-1.12, *P*-value = .72) (Figure 1, Table S1) analyses. We found no difference in the rate of selective cannulation in our unadjusted analysis of subgroups (Table 3).

**Table 3.** Procedure duration and rates of deep cannulation depending on time of day in patients with altered anatomy and procedures without trainee involvement.

	AM	PM	P-value
Altered anatomy	(*n* = 95)	(*n* = 106)	
Procedure duration (min)	57.3	46.0	***<.05***
Selective deep cannulation (%)	63.2	55.7	*.28*

No AEF involvement	(*n* = 147)	(*n* = 157)	
Procedure duration (min)	35.7	34.9	*.47*
Selective deep cannulation (%)	80.3	81.5	*.78*

### Secondary outcomes

We found no significant difference in the mean procedural duration between AM and PM procedures (33.7 min vs. 33.0 min *P*-value = .29) (Table 2). In the subgroup analysis of procedures performed on patients with altered anatomy, the mean procedural duration was higher in the AM group (57.3 min vs. 46.0 min *P*-value < .05) (Table 3).

Immediate bleeding occurred in 5.0% of AM procedures and 3.8% of PM procedures (*P*-value = .03) (Table 2). When adjusted for anticoagulant use, procedures performed in the AM were still more likely to result in immediate bleeding (OR 1.34, 95% CI, 1.04-1.73, *P*-value = .02) (Figure 1). We found no significant difference in rates of perforation in AM and PM procedures in both unadjusted (0.3% vs. 0.2% *P*-value = .53) (Table 2) or adjusted analysis (OR = 1.43, 95% CI, 0.46-4.86, *P*-value = .54) (Figure 1).

## Discussion

We report the largest review of 5672 ERCP’s performed over 10 years at a tertiary referral center. Our analysis demonstrates no significant differences in the rate of selective cannulation between procedures performed in the AM and PM. Rates of immediate adverse events were generally low.

Our findings correspond to those reported by the 2 prior studies evaluating the relationship between procedural timing and ERCP outcomes.^7,8^ Mehta et al. reviewed 296 procedures and concluded that there were no differences in selective cannulation rates, procedural completion rates, and adverse events in adjusted analysis between AM and PM ERCPs.^7^ This study was limited by a small sample size that was underpowered to ascertain any difference between these groups and only included patients with unaltered papilla anatomy.^7^ Similarly, Nielsen et al. evaluated 1006 ERCPs and compared those performed in the AM to those in the PM and determined no differences in adverse event rates between the 2 cohorts.^8^ Our study, however, identified a slightly higher rate of intraprocedural bleeding in the AM cohort. When contrasting the 2 cohorts, the baseline characteristics of the included patients and the procedural indications were similar. The only meaningful difference noted was the increased antithrombotic usage in the AM cohort. Although, when adjusted for anticoagulation use at baseline, the AM cohort still demonstrated a higher rate of intraprocedural bleeding. This finding challenges our hypothesis that afternoon procedures carry a higher risk of adverse events.

Our hypothesis that procedural timing, as a surrogate for physician fatigue and concentration, would affect outcomes in ERCP was motivated by studies in colonoscopy that posit an association between key quality metrics, such as ADR, and the timing of the procedure.^3^ The literature on this subject remains controversial with conflicting results from several studies.^13^ A recent and large systematic review and meta-analysis of studies investigating the impact of colonoscopy timing on key quality metrics, found that although there was no substantial difference in ADR between morning and afternoon colonoscopies, the polyp detection rate (PDR), was higher in the morning group.^14^

Colonoscopy, however, is inherently a different endoscopic procedure and is used most frequently as a screening tool compared to ERCP, which is a primarily therapeutic procedure.^15^ This important difference, in addition to the repetitive nature of the procedure, may make screening colonoscopy at higher risk for monotony which may reduce operator attentiveness over time. The heterogeneity of ERCP and the heightened risks associated with the procedure may be protective against fatigue and waning concentration. It is also important to highlight that St Michael’s hospital is a regional therapeutic endoscopy center with very high procedural volumes. In a meta-analysis of 4 comparative studies of ERCP outcomes in high- and low-volume centers, procedural success was favored in high-volume institutions.^16^ Thus, it is plausible that the procedural volume is protective against fatigue.

Our study has several limitations. Firstly, similar to the adenoma detection rate in colonoscopy, selective ductal cannulation is a key quality indicator in ERCP.^9^ We report a selective ductal cannulation rate of less than 90% which is lower than expected from a tertiary ERCP referral center. However, due to insufficient granularity of our data, we were unable to exclude patients where procedures were failed or altered due to sedation failure (which is estimated to be 5%-10% at our institution based on practice audit data), duodenal obstruction, or those that had been previously failed at another institution. Secondly, although the 2 comparator groups were well balanced on our reported baseline characteristics, important and unknown confounders cannot be accounted for.^17^ Thirdly, we did not use a screening tool to report fatigue and concentration, which may have been a better marker of fatigue than the surrogate of procedural timing. We were also unable to measure key outcomes in ERCP including delayed bleeding and post-ERCP pancreatitis, as we did not capture hospitalizations outside of St Michael’s hospital, thus would have missed encounters where patients presented to outside hospitals. Furthermore, our data includes instances where more than 1 endoscopists was performing ERCP in a given day and thus the morning operator may not always be the same as the afternoon operator. Though this is an acknowledged limitation, at our institution, endoscopists perform general and advanced endoscopic procedures throughout a given day, and thus we believe that the effect of endoscopy on concentration and fatigue to be present regardless of whether clinicians were solely performing ERCP’s or not. Finally, this was a single center study, at an institution with expertise in therapeutic endoscopy, which limits the generalizability of our results to other endoscopy units.

## Conclusions

In our single-center retrospective study, ERCP quality, including selective cannulation rates and immediate adverse events were not significantly different in procedures performed in the morning compared to those performed in the afternoon. Despite methodological limitations, our results support prior literature in demonstrating the efficacy and safety of afternoon compared to morning ERCP. Future studies should aim to utilize dedicated fatigue assessment tools and include low- and high-volume ERCP centers to further characterize the relationship between ERCP timing and outcomes.

## Supplementary material

Supplementary material is available at Journal of the *Canadian Association of Gastroenterology* online.

gwae028_suppl_Supplementary_material

gwae028_suppl_Supplementary_Coi_forms

## Data Availability

The data underlying this article cannot be shared publicly due to the privacy of individuals who participated in the study. The data will be shared on reasonable request to the corresponding author.
