# Supplementary material for: Outcomes of endoscopic retrograde cholangiopancreatography performed in the AM versus PM: does procedural timing matter?
Source: J Can Assoc Gastroenterol. 2024 Aug 26;7(6):411–5. doi: 10.1093/jcag/gwae028 (PMC11637997; doi:10.1093/jcag/gwae028)
Supplement: gwae028_suppl_Supplementary_material [file gwae028_suppl_supplementary_material.docx]

**Table S1. Multivariable logistic regression of deep biliary cannulation**

| **Predictor Variables** | **Odds ratio** | **95% CI** | **P-value** |
| --- | --- | --- | --- |
| Fellow operated | 1.07 | 0.78 – 1.43 | 0.67 |
| Altered anatomy | 0.29 | 0.21 – 0.39 | ***< 0.01*** |
| Conscious sedation used | 1.44 | 0.82 – 2.41 | 0.18 |
| AM procedure | 0.98 | 0.85 – 1.12 | 0.72 |
